# Broad Surveys of DNA Viral Diversity Obtained through Viral Metagenomics of Mosquitoes

**DOI:** 10.1371/journal.pone.0020579

**Published:** 2011-06-06

**Authors:** Terry Fei Fan Ng, Dana L. Willner, Yan Wei Lim, Robert Schmieder, Betty Chau, Christina Nilsson, Simon Anthony, Yijun Ruan, Forest Rohwer, Mya Breitbart

**Affiliations:** 1 College of Marine Science, University of South Florida, St. Petersburg, Florida, United States of America; 2 Department of Biology, San Diego State University, San Diego, California, United States of America; 3 Computational Science Research Center, San Diego State University, San Diego, California, United States of America; 4 Genome Institute of Singapore, Singapore; 5 Wildlife Disease Labs, San Diego Zoo's Institute for Conservation Research, San Diego, California, United States of America; University of Kansas Medical Center, United States of America

## Abstract

Viruses are the most abundant and diverse genetic entities on Earth; however, broad surveys of viral diversity are hindered by the lack of a universal assay for viruses and the inability to sample a sufficient number of individual hosts. This study utilized vector-enabled metagenomics (VEM) to provide a snapshot of the diversity of DNA viruses present in three mosquito samples from San Diego, California. The majority of the sequences were novel, suggesting that the viral community in mosquitoes, as well as the animal and plant hosts they feed on, is highly diverse and largely uncharacterized. Each mosquito sample contained a distinct viral community. The mosquito viromes contained sequences related to a broad range of animal, plant, insect and bacterial viruses. Animal viruses identified included anelloviruses, circoviruses, herpesviruses, poxviruses, and papillomaviruses, which mosquitoes may have obtained from vertebrate hosts during blood feeding. Notably, sequences related to human papillomaviruses were identified in one of the mosquito samples. Sequences similar to plant viruses were identified in all mosquito viromes, which were potentially acquired through feeding on plant nectar. Numerous bacteriophages and insect viruses were also detected, including a novel densovirus likely infecting *Culex erythrothorax*. Through sampling insect vectors, VEM enables broad survey of viral diversity and has significantly increased our knowledge of the DNA viruses present in mosquitoes.

## Introduction

Broad surveys of natural viral diversity are technically challenging due to the inability to sample a sufficient number of individuals from different host species and the difficulty of characterizing previously undescribed viruses. An effective strategy for exploring viral diversity would need to simultaneously identify a wide range of viral types in a large number of individuals. Since female mosquitoes draw blood from a wide range of vertebrate hosts including humans, non-human primates, other mammals and birds [1], and also feed on plant nectar, they effectively sample numerous important viral reservoirs. Here we describe the use of metagenomics to investigate viruses found in insect vectors and the hosts they feed upon. This method, called vector-enabled metagenomics (VEM), combines the power of metagenomics for discovering novel viruses with the natural ability of insect vectors to integrate viral diversity over space, time, and many hosts [2].

To date, the majority of mosquito virus studies have focused on the detection of specific, well-described RNA arboviruses [3], [4], less is known about the diversity of DNA viruses in mosquitoes. Viruses present in mosquitoes can include viruses that are biologically or mechanically transmitted by these vectors, as well as other viruses that are not transmitted by mosquitoes but are drawn indiscriminately from host reservoirs. Characterizing new viruses is difficult due to limitations of current detection methods [5]. Many viruses cannot be cultured in the laboratory, and methods such as degenerate PCR and pan-viral microarrays rely on the detection of highly conserved regions in known viral genomes for viral discovery [6]. To circumvent these issues, recent studies have demonstrated the effectiveness of viral particle purification and shotgun sequencing (viral metagenomics) for describing novel viruses [5], [7]. Using viral metagenomics, novel viruses have been characterized from nasopharyngeal aspirates [8], fecal samples [9], [10], blood [11], [12], and tissue samples such as lungs [13], [14] and tumors [15]. However, to date, no published studies have applied metagenomic sequencing to explore the diversity of viruses present in mosquitoes. In this study, we performed metagenomic sequencing on viruses purified from three mosquito samples from San Diego, California to provide a snapshot of the diversity of DNA viruses found in mosquitoes.

## Results and Discussion

### Novel and largely unexplored virus sequences in mosquitoes

**Figure 1:**
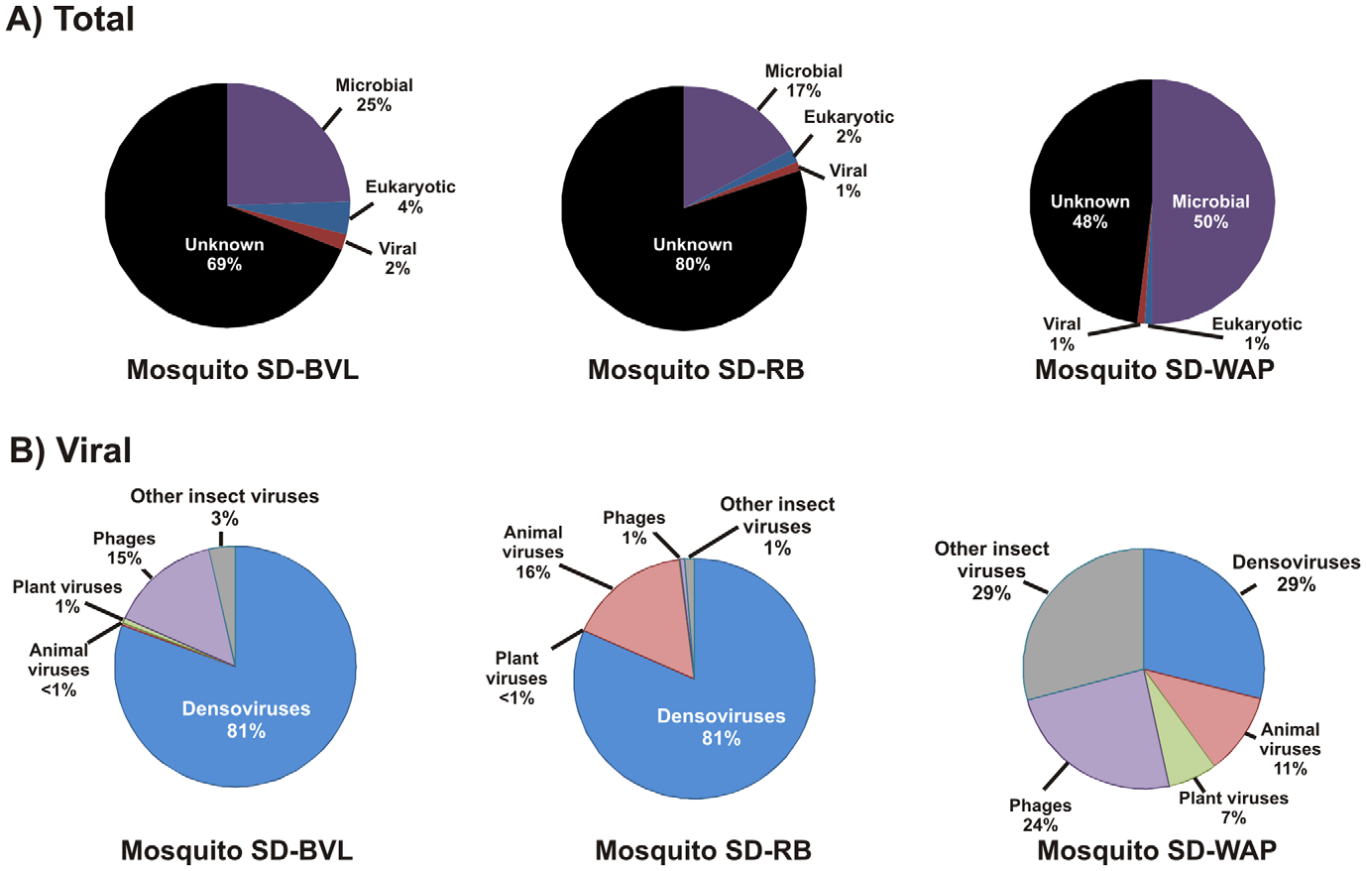
Taxonomic classification of the metagenomic sequences from the three mosquito viromes. A) Classification based on tBLASTx (E-value <0.001) against the Genbank non-redundant database. B) Breakdown of the viral sequences into four major categories: animal, plant, insect viruses (densoviruses and other insect viruses) and bacteriophages. Samples were obtained from 3 sites in San Diego: Buena Vista Lagoon (SD-BVL), River Bank (SD-RB), and Wild Animal Park (SD-WAP).

By performing viral metagenomics on mosquitoes, this study aimed to broadly survey the viruses present in the many hosts that mosquitoes feed upon. For each of the three mosquito samples, viruses were purified and approximately half a million sequences were generated from purified viral DNA (Table S1). Based on the most significant tBLASTx similarities, the mosquito viromes contained sequences related to a wide range of animal, insect, plant, and bacterial viruses (Fig. 1B). Sequences with nucleotide-level identity to previously described viruses were limited to mosquito densoviruses, human papillomavirus 23 (HPV23), and a few phages (95–100% identities, Tables S3 and S4). The majority of the virome sequences were completely unknown and most recognizable viral sequences had only low levels of similarity to known viruses (32–70% amino acid identity), suggesting a highly novel and diverse viral community sampled by the mosquitoes.

### Unclassified sequences likely represent novel viruses

The majority of the sequences in all mosquito viromes were completely unidentifiable based on sequence similarity (>48%, Fig. 1A), which is consistent with other viral metagenomic studies [12], [14], [15], [16]. This suggests that the reservoir of viruses in mosquitoes is novel and largely unexplored. Since sequencing was performed on purified virus particles, these divergent sequences likely originated from uncharacterized viral genomes. However, ongoing advancement of animal virus discovery can help elucidate the identities of these virus sequences found in mosquitoes. For example, when HPV112 was discovered from human skin in the past year [17], several contigs from this study that were previously classified as “unknown” were able to be recognized as papillomavirus sequences (see below). This example demonstrates that many of the unidentifiable sequences are likely represent novel viruses that are too divergent from known viruses to be recognized by sequence similarity searches. Increasing knowledge of animal and plant virus diversity has the potential to reveal the identities and hosts of these unknown viral sequences in the future.

### Distinct viromes of the three mosquito samples

**Figure 2:**
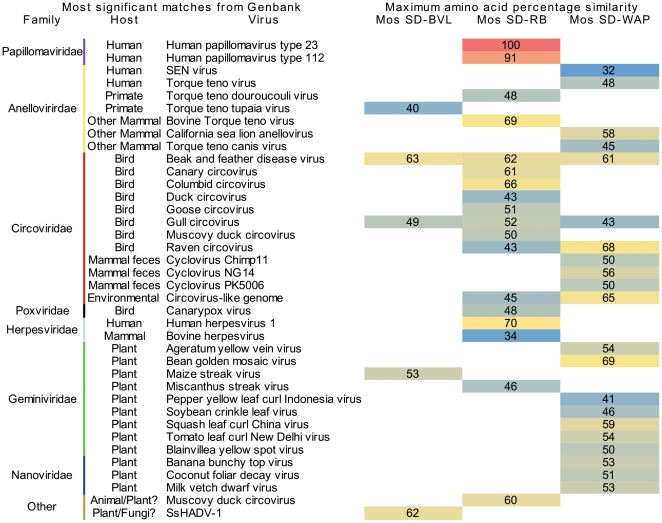
Classification of vertebrate and plant virus sequences present in the three San Diego mosquito viromes. The family, host, and name of the most significant tBLASTx similarities in the Genbank non-redundant database are shown, with the colors representing the level of amino acid identity.

Each mosquito virome contained a different complement of viruses based on several analyses. First, BLAST searches (Fig. 2, Table S2, S3 and S4) showed different viral types in each sample. Second, distinct viromes were supported by specific PCR showing that viruses could generally only be amplified from the sample where they were originally identified (Table S2). Two exceptions to this trend were the densoviruses, which were present in all samples, and Mosquito VEM Anellovirus – SDWAP B, which was detected in both of the samples examined by PCR. Finally, cross-BLASTn analysis was used to determine the percent of sequences shared between the three metagenomes (Table S5). Mosquito SD-RB and SD-WAP had few common sequences (5%), while each shared slightly more sequences with SD-BVL (12% and 11% respectively). Most sequences shared between samples were related to mosquito densoviruses, while unknown sequences and sequences similar to other viral genomes were less likely to be shared. This suggests that each mosquito virome shared a small core component, largely composed of insect viruses infecting mosquitoes. The larger component of the mosquito virome, consisting of animal, plant and bacterial viruses, is more variable, and thus distinct between samples.

The distance between mosquito sampling sites is 30–50 km (Fig. S1), which is far greater than the average flight range for a host-seeking *Culex erythrothorax* mosquito [18], [19]. The SD-WAP sample consisted exclusively of *C. erythrothorax* mosquitoes collected from an inland region of San Diego County in 2009, while both the SD-RB and SD-BVL mosquito samples consisted of mixed mosquito species drawn from coastal regions in 2006. Since mosquitoes draw blood within a radius of a few hundred meters, each sample likely contains viruses from animal hosts within mosquito's flight range. Although the three mosquito samples were of different species composition and were collected in different locations at different times, the distinct viromes demonstrate the diverse and heterogeneous nature of the viral community sampled by the mosquitoes.

### Animal viruses identified in mosquitoes

Contiguous sequences (contigs) assembled from the mosquito viromes had similarities to five families of animal viruses, namely *Anelloviridae*, *Circoviridae*, *Herpesviridae*, *Poxviridae* and *Papillomaviridae* (Fig. 2), that infect a wide range of hosts including humans, primates, other mammals, and birds. Although other mosquito species can be specific in the hosts they feed on, *Culex erythrothorax* feed on a variety of mammals and birds opportunistically [19], allowing them to sample viruses from many different animal hosts. Although this is not an exhaustive investigation of total animal virus diversity, metagenomics performed on insect vectors with broad host ranges provides a way to elucidate a portion of the pan-animal virome.

#### Papillomaviruses

**Figure 3:**
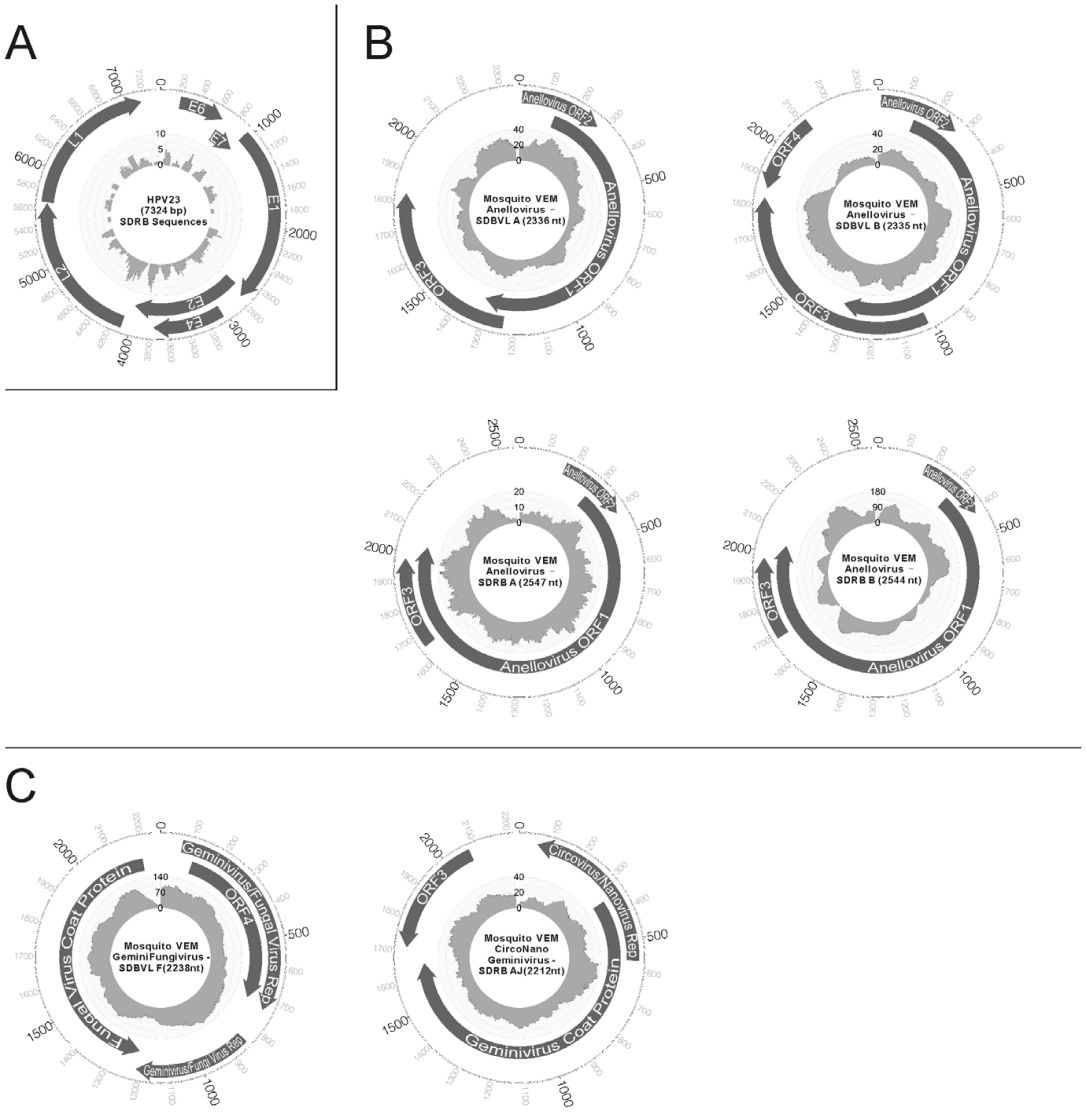
Genome organization and coverage of several putative virus genomes discovered in mosquito viromes. A) Human papillomavirus 23 (HPV23), B) Novel anelloviruses, C) Novel viruses with unique genome organization. Open reading frames are highlighted on the genome map and the amount of coverage from the metagenomic reads of the sample the virus was identified in is shown in the center.

**Figure 4:**
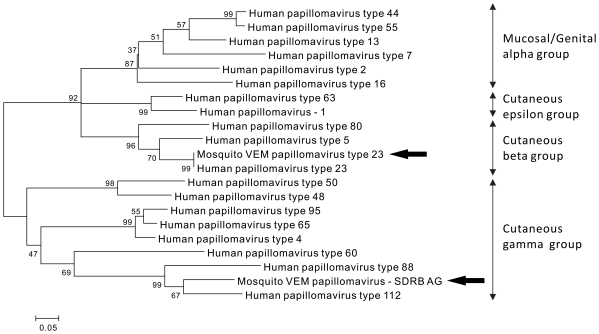
Neighbor joining tree based on the amino acid alignment of Mosquito VEM Papillomaviruses with the partial capsid protein L2 of representative HPV types. Mosquito VEM Papillomavirus – SDRB AF shared similarity to a different region of capsid protein L2, but produced identical tree topography (data not shown). Papillomavirus sequences identified in mosquito virome SD-RB are indicated by arrows, all of which belong to the cutaneous groups.

Sequences related to novel and previously described papillomaviruses were identified in the Mosquito SD-RB virome. Numerous sequences had >95% nucleotide identity to human papillomavirus type 23 (HPV23) (Table S4). Comparison with the HPV23 genome revealed near-complete coverage from the metagenomic sequences (Fig. 3). Additionally, sequences related to human papillomavirus type 112 (HPV112) were identified. Mosquito VEM Papillomavirus - SDRB AE shared 91% nucleotide identity to the E1 gene of HPV112. Mosquito VEM Papillomavirus SDRB AF and AG (MosVemPapAG) shared only 76% and 71% nucleotide identities respectively to the minor capsid protein L2 gene of HPV112, and did not have any significant nucleotide identities to any other HPV types. Phylogenetic analysis based on this partial minor capsid protein region showed that MosVemPapAG is most closely related to HPV112, and belongs to the cutaneous gamma-papillomavirus genus (Fig. 4). Although we cannot confirm the host of MosVemPapAG, it groups phylogenetically with other human papillomaviruses. All papillomavirus sequences identified in the mosquitoes belonged to the cutaneous groups (beta and gamma groups), suggesting that mosquitoes may acquire papillomaviruses from the host's skin during feeding.

Although more than 80% of normal human skin harbors papillomaviruses [20], human papillomaviruses have not previously been described in mosquitoes. This study is the first demonstration of sequences related to a human papillomavirus (HPV23) in mosquitoes, and also provides evidence that mosquitoes can harbor novel papillomaviruses. Papillomaviruses were only identified in one of the mosquito samples, suggesting that these viruses may only be present in mosquitoes sporadically. It was previously noticed that mosquitoes can transmit rabbit papillomavirus [21]; however, further research is needed to determine the prevalence and transmission potential of different types of human papillomaviruses in mosquitoes.

#### Anelloviruses and Circoviruses

**Figure 5:**
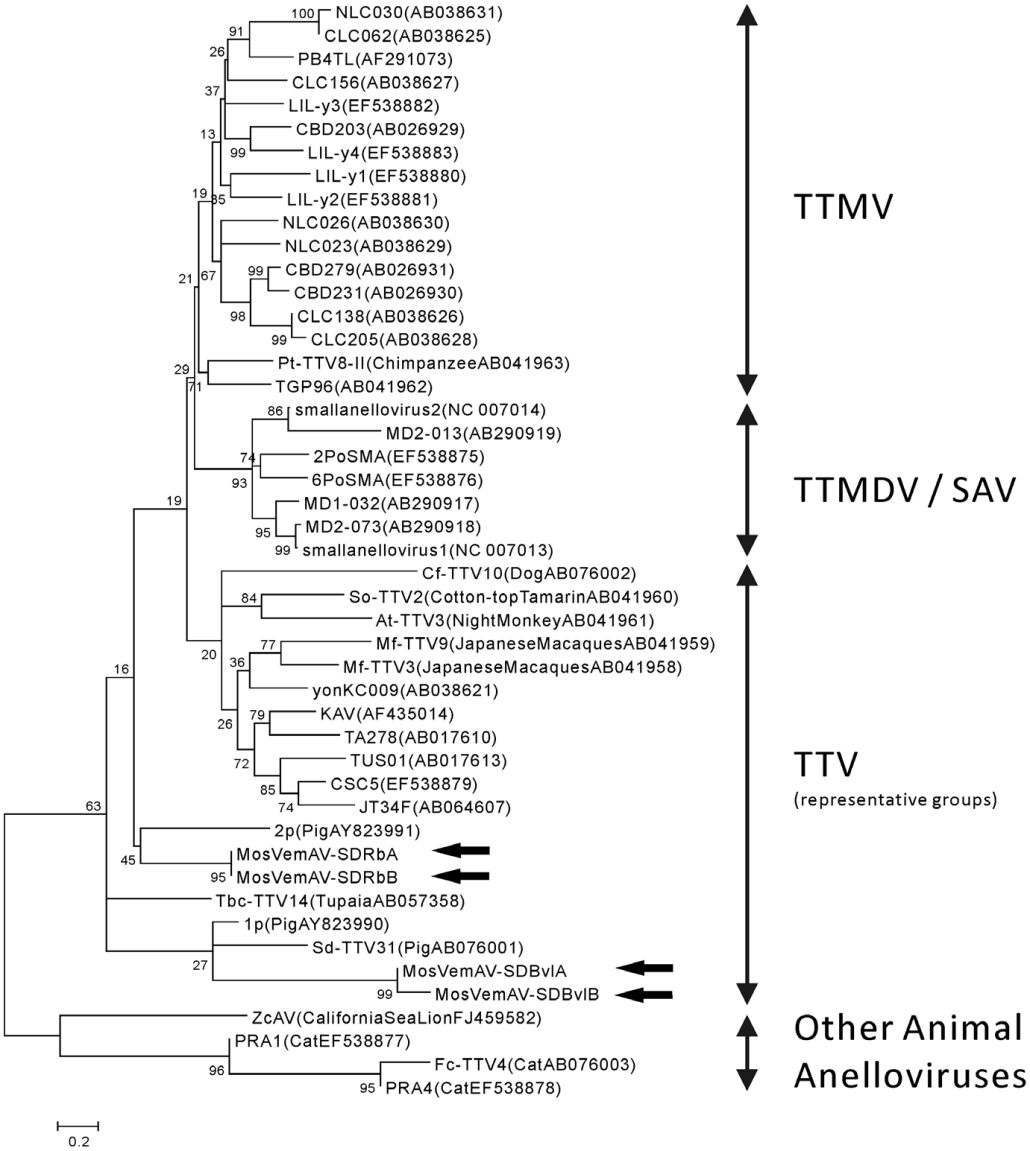
Neighbor joining phylogenetic tree of anelloviruses constructed using the entire nucleotide sequence of ORF1. Genbank accession numbers are shown in parentheses, and the hosts are indicated for any non-human sequences. The newly discovered anelloviruses from the mosquito viromes are indicated by arrows.

All anellovirus sequences identified in the viromes were novel (<70% amino acid identity to known anelloviruses, Fig. 2 and Table S2), suggesting that the animal hosts the mosquitoes feed on contain largely uncharacterized anellovirus diversity. The complete genomes of four putative viral genomes were sequenced (Fig. 3). Phylogenetic analysis based on the complete nucleotide sequence of ORF1 placed SD-BVL and SD-RB anelloviruses into the Torque teno virus (TTV) group, but forming a distinct genetic lineage from other TTV sequences (Fig. 5). Anellovirus genomes from an individual sample were closely related to each other, but unique genomes were found in different mosquito samples. In addition to the complete genomes, partial contigs with similarity to bovine TTV, human TTV, and human SEN virus were identified (Fig. 2 and Table S2).

A diverse range of circoviruses was identified in the mosquito samples (Fig. 2 and Table S2). No significant pairwise nucleotide identity was shared between the replication genes of any of the circoviruses (data not shown), suggesting that these contigs represent distinct viral genomes. PCR assays for SD-BVL circovirus sequences were positive in the sample they originated from, but negative in the SD-WAP sample (Table S2), suggesting distinct circoviruses were present in each virome.

These results demonstrate that the pan-animal virome contains diverse and largely uncharacterized circoviruses and anelloviruses, which mosquitoes may routinely obtain from viremic hosts during blood feeding. Viruses belonging to the *Circoviridae* and *Anelloviridae* families contain small, circular, single-stranded DNA genomes, and are usually identified in blood [22], [23]. Circoviruses are known to infect birds and pigs [24], and diverse circoviruses have been identified in aquatic environments [16], [25], [26], as well as in human, chimpanzee and bat feces [27], [28], [29], [30].The identification of sequences similar to avian circoviruses in mosquitoes is interesting because birds are the reservoir and secondary amplifying hosts of many mosquito-transmitted arboviruses, such as Eastern, Western, Japanese and St Louis equine encephalitis virus and West Nile virus [31]. Anelloviruses are known to infect humans, non-human primates, domestic animals and marine mammals [13], [32], [33], [34], but the pathology of anelloviruses remains unknown [33], [35].

#### Herpesvirus-like and Poxvirus-like sequences

In sample SD-RB, four contigs with amino-acid-level sequence similarities to herpesviruses and poxviruses were identified (Fig. 2 and Table S2). However, these contigs are only short portions of the genomes so it is impossible to determine more details about their identities or hosts.

### Plant viruses identified in mosquitoes

#### Geminiviruses and Nanoviruses

Sequences with similarities to plant viruses were consistently identified in the mosquito viromes (Fig. 2 and Table S2). All three viromes contained sequences related to geminiviruses, and sample SD-WAP had sequences related to nanoviruses. Mosquitoes are known to feed on plant nectar, indicating a potential source of these viruses. However, no plant viruses have been previously described in mosquitoes, so the ability of mosquitoes to transmit plant viruses still needs to be investigated through laboratory and field-based transmission studies. Other insect vectors that feed on plants, such as whiteflies, are known to transmit a diversity of plant viruses [36]. In a related study using VEM to examine the viral community in whiteflies, almost all of the viral sequences shared high levels of nucleotide identity with previously described plant geminiviruses (Ng *et al.* in review). In contrast, the plant virus sequences in the mosquito viromes showed only weak amino acid level identities to known viruses (46%–53%; Fig. 2). These sequences from mosquitoes may represent extremely novel plant viruses, or could be part of recombinant genomes infecting other hosts.

### Insect viruses identified in mosquitoes

#### Parvoviridae and Poxviridae

**Figure 6:**
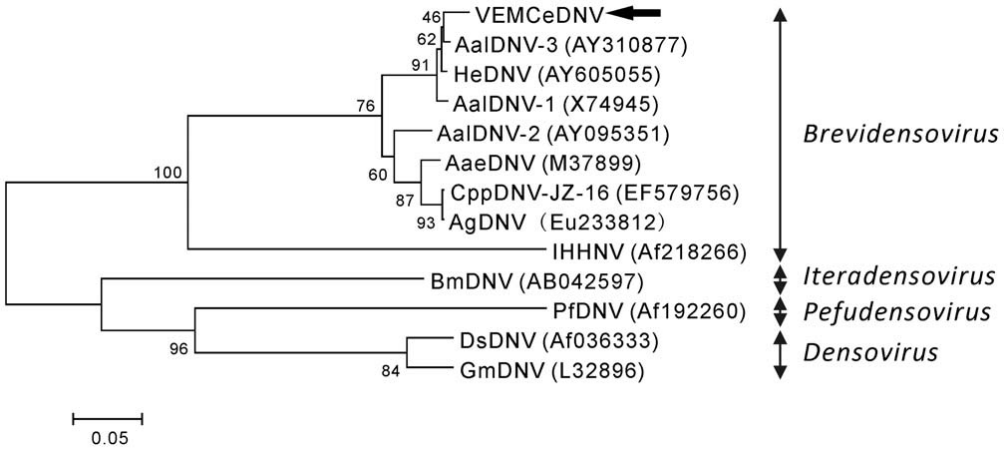
Neighbor joining phylogenetic tree of VEMCeDNV and other densoviruses based on alignment of the 720-bp partial NS1 gene nucleotide sequences. The VEMCeDNV from the *C. erythrothorax* mosquitoes in sample SD-WAP is indicated with an arrow.

A diverse range of insect viruses was identified in the mosquito viromes (Table S3 and S4). The majority of the sequences were similar to mosquito densoviruses (DNVs), specifically *Aedes albopictus* densovirus (AalDNV) and *Haemogogus equinus* densovirus (HeDNV). Since *H. equinus* and *A. albopictus* mosquitoes are not indigenous to San Diego, these sequences most likely represent densoviruses that infect the sampled mosquito species, primarily *C. erythrothorax*. Using PCR targeting the NS1 gene region, we further investigated the presence of densoviruses in the SD-WAP sample, which contained exclusively *C. erythrothorax* mosquitoes. The 720 base pair sequence of the PCR product (Accession #GU810839) was closely related (96% nucleotide identity) to HeDNV (Fig. 6). This sequence (VEM Culex erythrothorax densovirus; VEMCeDNV) most likely represents a new mosquito densovirus that infects *C. erythrothorax* mosquitoes.

Densoviruses have been detected frequently in mosquito cell lines, and more rarely in wild-caught mosquitoes, where they are perpetuated through both horizontal and vertical transmission [37], [38], [39]. Densovirus infection is highly lethal in cell lines and early stage larvae, however, infection at the late stages of larval development generally leads to a persistent and transmissible viremia [37], [39]. Mosquito densoviruses are stable vectors for transformation of mosquitoes [40], [41], [42], which has created interest in using these viruses for mosquito and malaria control, either directly as lethal agents or as possible carriers of transgenes [38]. Viral paratransgenesis takes advantage of the densoviruses to introduce genes that are lethal to mosquitoes or the pathogens that they carry. Viral paratransgenesis efforts can greatly benefit from the discovery of new densoviruses, such as those identified in this study. *C. erythrothorax* is the most common mosquito in San Diego County, and is suspected to be an emergent vector of West Nile Virus [43]. Further studies of VEMCeDNV will be necessary to determine its efficacy as a biocontrol agent for *C. erythrothorax*.

Many other insect viruses were also identified, but none were found in all three samples (Table S3). Sample SD-BVL had the highest diversity of densoviruses, followed by SD-RB, then by SD-WAP (Table S3). Many sequences showed less than 85% amino acid identities to known densoviruses and unclassified insect viruses (Table S3), suggesting the presence of many novel insect viruses in mosquitoes.

### Phages identified in mosquitoes

The mosquito viromes contained a large diversity of phage sequences (Table S3), including members from *Myoviridae*, *Podoviridae*, and *Siphoviridae*, as well as unclassified phages. Most of the phage sequences found in the mosquitoes only shared amino acid identities to known phages. However, in sample SD-RB, numerous sequences had 100% nucleotide identities to Propionibacterium phage PAD42 and PA6, Acyrthosiphon pisum secondary endosymbiont phage (APSE) 1–6, and Enterobacteria phage lambda (Table S3), suggesting that these known phages (or closely related phages) were present in the mosquito SD-RB virome. The three mosquito viromes differed in terms of the types of phages with BLAST similarities (Table S3), suggesting that each sample had a distinct phage content.

Phages identified in the mosquito viromes may infect the bacterial flora of the mosquito or that of the hosts they have fed upon. *Propionibacterium acnes*, the host for Propionibacterium phage PAD42 and PA6, is a commensal bacterium of human skin, so it is possible that mosquitoes acquire this bacterium and its phages during blood feeding [43]. Sequences related to Enterobacteria phage might originate from the digestive system of mosquitoes and sequences with identities to phage APSE might infect endosymbiotic bacteria of mosquitoes. Phage APSE-1 through 6 infect *Hamiltonella defensa*, an endosymbiont of aphids and other sap-feeding insects that protects the aphids from wasp attack by killing the developing wasp larvae [44]. Phage APSE-3 carries a toxin-encoding gene that provides the endosymbiont with defense against wasp larvae [45], and other APSE phages are also known to encode toxin genes [46], [47]. Mosquitoes are not known to be hosts for parasitic wasps, but endosymbiotic bacteria such as *Wolbachia* are known to infect mosquitoes and interfere with the reproductive biology of their host through cytoplasmic incompatibility [48]. Identification of sequences with high nucleotide identity to Phage APSE in this study suggests that mosquitoes potentially harbor other endosymbiotic bacteria and their phages, possibly to increase mosquito survivorship, or that of their eggs, through deterring predation.

This is the first study demonstrating the presence of a broad range of phages in mosquitoes, and distinct phage profiles for each mosquito virome. A diversity of phages with different inferred bacterial hosts was observed in the mosquitoes. Surveys based on the 16S rRNA gene support the notion of high bacterial diversity in mosquitoes [49], but to date, no metagenomic studies have examined the bacterial communities associated with mosquitoes. Future investigation of the role of bacteria and phages in mosquitoes is important, as they likely affect the host's physiology and fitness.

### Viruses with unique genome organizations identified in mosquitoes

Two complete genomes were identified that contained features from different virus families. In sample SD-RB, genome Mosquito VEM CircoNanoGeminivirus - SDRB AJ showed a combination of features from the *Circoviridae* (animal virus), *Nanoviridae* (plant virus) and *Geminiviridae* (plant virus) families (Fig. 3). ORF2 of this virus showed similarity to the pfam viral replication protein 02407 superfamily, and had BLASTp hits to the replication protein from both the *Circoviridae* (29% amino acid identity to Porcine circovirus 2) and *Nanoviridae* (44% amino acid identity to Faba bean necrotic yellows virus) families. ORF1 of this virus showed similarity to the pfam geminivirus coat protein 00844 superfamily, and had 27% amino acid identity to the geminivirus Eragrostis curvula streak virus.

In sample SD-BVL, genome Mosquito VEM GeminiFungivirus - SDBVL G (Fig. 3) shared features from both the single-stranded DNA (ssDNA) plant virus *Geminiviridae* family and the single-stranded DNA (ssDNA) fungal virus, Sclerotinia sclerotiorum hypovirulence associated DNA virus (SSHADV1) [50]. Protein conserved domain searches on ORF2 revealed similarity to the geminivirus replication catalytic domain pfam 00799, and a BLASTp search showed 28% amino acid identity to the geminivirus Tomato mottle leaf curl virus replication protein and 34% amino acid identity to the fungal virus SSHADV1. ORF1 showed 32% amino acid identity to the SSHADV1 coat protein. ORF3 of this virus had 41% amino acid identity to the replication-associated protein from the geminivirus Eragrostis curvula streak virus, and 62% amino acid identity to the replication-associated protein from SSHADV1.

### VEM as an effective method for exploring viral diversity

Understanding total viral diversity is important for animal and human health. However, broad surveys of animal viral diversity is difficult due to the large number of individuals to sample, as well as the methodological limitations in characterizing novel viruses. The unique approach described here circumvents these issues by allowing the discovery of multiple novel viruses from many hosts in a single experiment. Through the use of VEM, this study created a baseline of the DNA virus community present in mosquitoes, shedding light on the high diversity of animal, plant, insect, and bacterial viruses that are present in this important vector. Although the discovery of a viral sequence does not always indicate active infection, the initial characterization will enable future studies to investigate viral prevalence and link viruses to hosts and disease symptoms. The application of this technique to mosquitoes from other regions, as well as other types of vectors, will greatly enhance our understanding of viral diversity.

VEM is a versatile technique that can be further refined to answer specific questions. Instead of purifying viruses from whole mosquitoes, VEM can be performed on dissected blood meals, surveying viruses specifically from the animal blood and plant nectar that the mosquitoes feed on and excluding viruses that may be present on the outside of the mosquitoes. Similarly, performing metagenomics on viruses purified from the dissected mosquito salivary glands, or mosquito saliva emitted during sugar feeding [51] can identify arboviruses with potential for transmission to animals. VEM can also be performed to characterize RNA viruses through the addition of a random-primed reverse transcription step [52]. Finally, the multiple displacement amplification used in this study is known to preferentially amplify small ssDNA circular genomes [53], [54], [55], and the identifiable sequences in this study were dominated by mosquito densoviruses. To identify more double-stranded DNA (dsDNA) viruses, alternate amplification methods without this bias could be used, or ssDNA could be selectively removed from the mosquito samples by Mung Bean nuclease treatment.

In conclusion, this study utilized VEM to demonstrate the presence of a highly novel and diverse reservoir of animal, plant, insect, and bacterial viruses present in mosquitoes. The three different mosquito viromes contained distinct virus profiles, showing heterogeneity in the circulating viral community. By enabling broad surveys of viral diversity from many hosts, the VEM approach described here will be transformative for our understanding of the ecology of animal, plant, insect, and bacterial viruses.

## Materials and Methods

### Sample collection

Three mosquito samples (Table S1) were collected from San Diego County, CA, USA (Fig. S1) using an EVS CO_2_ trap baited with dry ice (BioQuip Products, Inc., Rancho Dominguez, CA, USA). SD-BVL and SD-RB mosquito samples were killed by freezing at −80°C, while the SD-WAP mosquitoes were anesthetized with triethylamine and stored at 4°C. Mosquitoes were homogenized in 5 ml of suspension medium (SM) buffer using a Tissumizer (Tekmar Control Systems, Inc., Vernon, Canada) at 5,000–8,000 rpm. Mosquito debris was pelleted by two rounds of centrifugation at 1,500 *xg* at 4°C for 30 min.

### Viral particle purification and metagenomic sequencing

The protocol for viral particle concentration and purification was modified from previous studies [12], [13], [56], and an overview is shown in Table S6. Supernatants from the mosquito homogenates were filtered through a 0.45 µm syringe filter unit (Millipore, Billerica, MA) and viral particles were purified from the filtrate using a cesium chloride (CsCl) step gradient. The purified viral concentrate was examined by epifluorescence microscopy to verify the presence of viral particles, and ensure the absence of contaminating bacterial and eukaryotic cells [57]. The viral fraction was concentrated and washed twice with sterile SM buffer on a Microcon 30 column (Millipore), followed by treatment with 0.2 volumes of chloroform for 10 minutes, then incubation with 2.5 U DNase I per µl for 3 hours at 37°C.

Total DNA was extracted using a CTAB/Formamide protocol [58]. Extracted viral DNA was amplified using Genomiphi for 1.5 hours (GE Healthcare, Piscataway, NJ) based on the manufacturer's instructions. Following amplification, samples SD-BVL and SD-RB were sequenced with 454 GS20 pyrosequencing and sample SD-WAP was sequenced using 454 GS FLX technology. Longer read length in the SD-WAP virome resulted in an increased proportion of sequences with known identities compared to the other two viromes. The NCBI genome project numbers for the three viromes are 28413, 28467, and 49713.

### Bioinformatics

Metagenomic sequences were filtered using PRINSEQ [59] to remove short reads, and were compared to the GenBank non-redundant database using BLASTn and tBLASTx [60], [61]. Each sequence was assigned top-level taxonomy (Eukarya, Bacteria, Archaea, or Virus) based on its closest BLAST similarity (E-value <10^−3^ (Fig. 1A). Sequences with a best BLAST similarity to cellular genomes over ≥50 nt with an E-value <10^−3^ were removed prior to further classification. Remaining sequences were further classified based on best BLAST similarities to viral genomes (Fig. 1B). A cross-BLAST approach [14] was used to evaluate the similarity between mosquito metagenomes, where all sequences with BLASTn E-value <10^−5^ and ≥98% identity were considered shared.

Metagenomic sequences were assembled into contigs using the SeqMan Pro-assembler (DNASTAR, Madison, WI) with match size  = 35, minimum match percentage  = 95%, match spacing  = 15, maximum mismatch end bases  = 0. Contigs were compared to the non-redundant database using tBLASTx (E-value <0.001 [60], [61] and contigs representing complete genomes were manually analyzed using SeqBuilder (DNASTAR). For complete genomes, open reading frames (ORFs) were analyzed and annotated using Artemis [62] and BLASTn and BLASTp were performed to determine identity. The HPV sequences were also assembled to the HPV23 genome (Genbank Accession # U31781) as a reference using Sequencher 4.7 (Gene Codes, Ann Arbor, MI). Densovirus sequences were assembled into contigs using the 454 GS *De Novo* Assembler (Branford, CT). Viral contigs were deposited to Genbank under the accession number HQ335010-HQ335087.

### PCR screening

Nucleic acids from Mosquito SD-BVL and SD-WAP were amplified by Genomiphi (GE Healthcare), then subjected to PCR with primers designed based on selected contigs from the viromes (Table S7). Sample Mosquito SD-RB was unavailable for PCR testing. PCR primers were designed to amplify large regions of the contigs, encompassing many individual metagenomic sequence reads. PCR products were sequenced to verify the accuracy of the assemblies.

### Phylogenetic analysis

Alignments were performed using ClustalW multiple alignment in Bioedit [63], and MEGA4 was used for phylogenetic analysis with a neighbor joining method and bootstrap with 1000 replications [64]. Phylogenetic analysis for the papillomaviruses was based on the partial alignment of the minor capsid protein L2 sequence with representative HPVs. The phylogenetic analysis of the anelloviruses was based on alignment of the ORF1 nucleotide sequence with major anellovirus groups [13], [32]. For the densoviruses, the phylogenetic analysis was based on the nucleotide alignment of the partial NS1 gene PCR sequence with representative densoviruses [65].

## Supporting Information

Figure S1 Locations of mosquito samples, produced using Google Earth (http://earth.google.com/). Samples were obtained from 3 sites in San Diego: Buena Vista Lagoon (SD-BVL), River Bank (SD-RB), Wild Animal Park (SD-WAP).
(PDF)

Table S1 Sample description.
(PDF)

Table S2 Contigs and genomes with significant tBLASTx similarities to known vertebrate and plant viruses. The virus name, family and host of the most significant tBLASTx sequence in Genbank are shown. Complete genomes are indicated with asterisks (*). For PCR results, “Y” indicates that the contig was detected in that specific sample and “N” indicates that it was not.
(PDF)

Table S3 Analysis of the contigs with amino acid identities (tBLASTx, evalue<0.001) to bacteriophages and insect viruses.
(PDF)

Table S4 BLASTn analysis of the contigs with nucleotide identities to members of Papillomaviridae and Parvoviridae.
(PDF)

Table S5 Cross-BLASTn analysis of the mosquito metagenomes. A sequence was considered to be shared by two metagenomes if each sequence was the best BLASTn similarity for the other when the two metagenomes were compared with BLASTn.
(PDF)

Table S6 Overview of the VEM methodology for obtaining viral metagenomes from mosquitoes.
(PDF)

Table S7 Primers used in this study.
(PDF)
