# Supplementary material for: Broad Surveys of DNA Viral Diversity Obtained through Viral Metagenomics of Mosquitoes
Source: PLoS One. 2011 Jun 6;6(6):e20579. doi: 10.1371/journal.pone.0020579 (PMC3108952; doi:10.1371/journal.pone.0020579)

Fig. S1. Locations of mosquito samples, produced using Google Earth (http://earth.google.com/). Samples were obtained from 3 sites in San Diego: Buena Vista Lagoon (SD-BVL), River Bank (SD-RB), Wild Animal Park (SD-WAP).

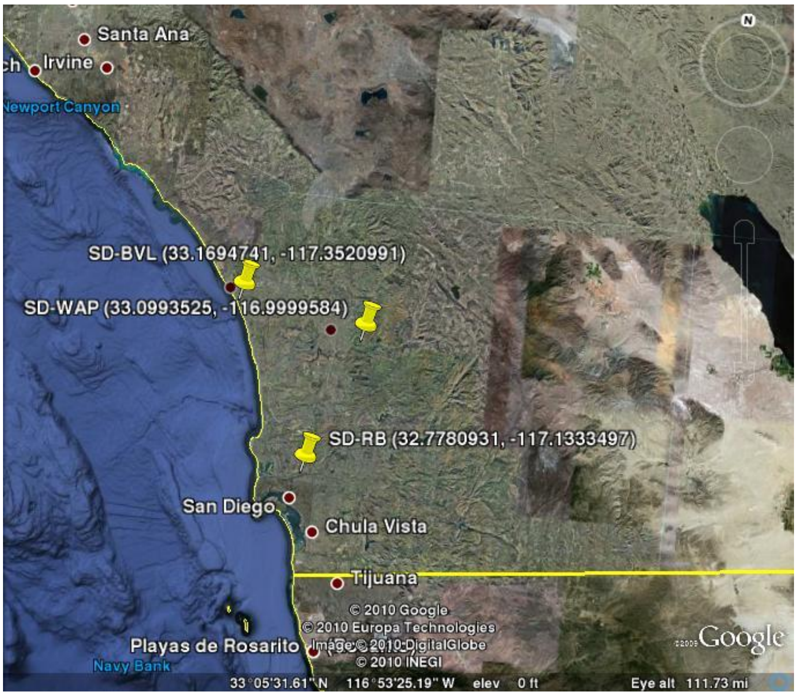

Supplement: Figure S1 — Locations of mosquito samples, produced using Google Earth (http://earth.google.com/). Samples were obtained from 3 sites in San Diego: Buena Vista Lagoon (SD-BVL), River Bank (SD-RB), Wild Animal Park (SD-WAP). (PDF) [file pone.0020579.s001.pdf]
