# Supplementary material for: Broad Surveys of DNA Viral Diversity Obtained through Viral Metagenomics of Mosquitoes
Source: PLoS One. 2011 Jun 6;6(6):e20579. doi: 10.1371/journal.pone.0020579 (PMC3108952; doi:10.1371/journal.pone.0020579)
Supplement: Table S1 — Sample description. (PDF) [file pone.0020579.s002.pdf]

Table S1. Sample description.

**Sample Name**	**Sampling Location (GPS)**	**Sampling Date**	**Sample composition**	**Sequencing Platform**	**# of Reads**	**Average Read Length (bp)**	**# of Bases**
Mosquito SD-BVL	Buena Vista Lagoon, SD, USA (33.17N,-117.35W)	01/25/06	80 female mixed species mosquitoes	GS20	340,098	102.61	35 Mb
Mosquito SD-RB	River Bank, SD, USA (32.78N, -117.13W)	06/06/06	200 female mixed species mosquitoes	GS20	615,576	104.16	64 Mb
Mosquito SD-WAP	Wild Animal Park, SD, USA (33.10N, -117.00W)	09/10/09	200 female *Culex erythrothorax* mosquitoes	GSFLX Titanium	259,591	398.78	104 Mb
