# Supplementary material for: Broad Surveys of DNA Viral Diversity Obtained through Viral Metagenomics of Mosquitoes
Source: PLoS One. 2011 Jun 6;6(6):e20579. doi: 10.1371/journal.pone.0020579 (PMC3108952; doi:10.1371/journal.pone.0020579)
Supplement: Table S2 — Contigs and genomes with significant tBLASTx similarities to known vertebrate and plant viruses. The virus name, family and host of the most significant tBLASTx sequence in Genbank are shown. Complete genomes are indicated with asterisks (*). For PCR results, “Y” indicates that the contig was detected in that specific sample and “N” indicates that it was not. (PDF) [file pone.0020579.s003.pdf]

Table S2. Contigs and genomes with significant tBLASTx similarities to known vertebrate and plant viruses. The virus name, family and host of the most significant tBLASTx sequence in Genbank are shown. Complete genomes are indicated with asterisks (*). For PCR results, "Y" indicates that the contig was detected in that specific sample and "N" indicates that it was not.

Genome / contig name	Contig length	Sample	PCR result using		Most significant matches from Genbank (tBLASTx, E-value < 0.001)			
			Sample SD-BVL	Sample SD-WAP	Family	Host	Virus (E-value)	% a.a. identity
Mosquito VEM Anellovirus - SDBVL A	2336*	SD-BVL	Y	N	Anellovirirdae	Primate	Torque teno tupaia virus (2e-47)	40
Mosquito VEM Anellovirus - SDBVL B	2335*	SD-BVL	Y	N	Anellovirirdae	Primate	Torque teno tupaia virus (6e-46)	40
Mosquito VEM Circovirus - SDBVL C & D	236 & 962	SD-BVL	Y	-	Circoviridae	Bird	Beak and feather disease virus (8e-008 & 9e-034)	63 & 62
Mosquito VEM Circovirus - SDBVL E	327	SD-BVL	Y	-	Circoviridae	Bird	Gull circovirus (2e-012)	49
Mosquito VEM Geminivirus - SDBVL F	194	SD-BVL	Y	-	Geminiviridae	Plant	Maize streak virus(4e-005)	53
Mosquito VEM GeminiFungivirus - SDBVL G	2238*	SD-BVL	Y	N	Unclassified recombinant virus	Plant/ Fungi?	Sclerotinia sclerotiorum hypovirulence associated DNA virus 1 (3e-76)	62
Mosquito VEM Anellovirus - SDRB A	2547*	SD-RB	N	N	Anellovirirdae	Primate	Torque teno douroucouli virus (2e-28)	43
Mosquito VEM Anellovirus - SDRB B	2544*	SD-RB	N	N	Anellovirirdae	Primate	Torque teno douroucouli virus (2e-30)	48
Mosquito VEM Anellovirus - SDRB C	500	SD-RB	-	-	Anellovirirdae	Mammal	Bovine Torque teno virus (3e-004)	69
Mosquito VEM Circovirus - SDRB D & E	699 & 183	SD-RB	-	-	Circoviridae	Bird	Beak and feather disease virus (1e-014 & 8e-004)	50 & 52
Mosquito VEM Circovirus - SDRB F - L	196-1626	SD-RB	-	-	Circoviridae	Bird	Columbid circovirus (3e-004 - 8e-017)	41-66
Mosquito VEM Circovirus - SDRB M	417	SD-RB	-	-	Circoviridae	Bird	Goose circovirus (6e-05) (2nd best hit)	51
Mosquito VEM Circovirus - SDRB N & O	753 & 557	SD-RB	-	-	Circoviridae	Bird	Gull circovirus (2e-008 & 2e-009)	47 & 52
Mosquito VEM Circovirus - SDRB P	410	SD-RB	-	-	Circoviridae	Bird	Muscovy duck circovirus (4e-004)	50
Mosquito VEM Circovirus - SDRB Q & R	863 & 261	SD-RB	-	-	Circoviridae	Bird	Raven circovirus (1e-007 - 8e-006)	34 - 43
Mosquito VEM Circovirus - SDRB S & T	988 & 850	SD-RB	-	-	Circoviridae	Bird	Duck circovirus (2e-11 & 7e-19) (2nd best hit)	43
Mosquito VEM Circovirus - SDRB U	463	SD-RB	-	-	Circoviridae	Bird	Canary circovirus (8e-005)	61
Mosquito VEM Circovirus - SDRB V & X	485 & 551	SD-RB	-	-	Circoviridae	Environmental	Circovirus-like genome RW-C (1e-05)	41 - 45
Mosquito VEM Geminivirus - SDRB AA	915	SD-RB	-	-	Geminiviridae	Plant	Miscanthus streak virus (5e-014)	46
Mosquito VEM Herpesvirus - SDRB AB	184	SD-RB	-	-	Herpesviridae	Human	Human herpesvirus 1 (2e-005)	70
Mosquito VEM Herpesvirus - SDRB AC	742	SD-RB	-	-	Herpesviridae	Mammal	Bovine herpesvirus (3e-04)	34
Mosquito VEM Papillomavirus - SDRB AD	Many	SD-RB	N	N	Papillomaviridae	Human	Human papillomavirus type 23 (3e-117)	~100
Mosquito VEM Papillomavirus - SDRB AE - AG	152 - 254	SD-RB	N	N	Papillomaviridae	Human	Human papillomavirus type 112 (6e-19 - 5e-12)	67-80
Mosquito VEM Poxvirus - SDRB AH & AI	391 & 380	SD-RB	-	-	Poxviridae	Bird	Canarypox virus (5e-07 & 2e-06)	45 & 48
Mosquito VEM CircoNanoGeminivirus - SDRB AJ	2212*	SD-RB	N	N	Unclassified recombinant virus	Animal/ Plant?	Muscovy duck circovirus (3e-25)	60
Mosquito VEM Anellovirus - SDWAP A	1969	SD-WAP	-	-	Anellovirirdae	Human	SEN virus (1e-05)	32
Mosquito VEM Anellovirus - SDWAP B	2594	SD-WAP	Y	Y	Anellovirirdae	Human	Torque teno virus (8e-10)	36
Mosquito VEM Anellovirus - SDWAP C - E	283 - 591	SD-WAP	-	-	Anellovirirdae	Human	Torque teno virus (2e-04 - 2e-10)	44 - 48
Mosquito VEM Circovirus - SDWAP F	429	SD-WAP	-	-	Circoviridae	Mammal	Cyclovirus Chimp11 (7e-08)	50
Mosquito VEM Circovirus - SDWAP G	586	SD-WAP	-	-	Circoviridae	Bird	Beak and feather disease virus (9e-10)	61
Mosquito VEM Circovirus - SDWAP H & I	695 & 1980	SD-WAP	-	-	Circoviridae	Bird	Gull circovirus (1e-17 & 2e-26)	43 & 53
Mosquito VEM Circovirus - SDWAP J	2778	SD-WAP	-	-	Circoviridae	Bird	Raven circovirus (8e-18) (2nd best hit)	68
Mosquito VEM Circovirus - SDWAP K	942	SD-WAP	-	-	Circoviridae	Mammal	Cyclovirus NG14, complete genome (3e-06)	56
Mosquito VEM Circovirus - SDWAP L	1998	SD-WAP	-	-	Circoviridae	Environmental	Circovirus-like genome RW-E (2e-79)	65
Mosquito VEM Circovirus - SDWAP M	1354	SD-WAP	-	-	Circoviridae	Environmental	Circovirus-like genome CB-A (2e-07)	37
Mosquito VEM Circovirus - SDWAP N	875	SD-WAP	-	-	Circoviridae	Environmental	Cyclovirus PK5006 (2e-26) (2nd best hit)	50
Mosquito VEM Nanovirus - SDWAP O	1151	SD-WAP	-	-	Nanoviridae	Plant	Banana bunchy top virus (5e-10)	53
Mosquito VEM Nanovirus - SDWAP P	1265	SD-WAP	-	-	Nanoviridae	Plant	Milk vetch dwarf virus (1e-09)	53
Mosquito VEM Nanovirus - SDWAP Q	510	SD-WAP	-	-	Nanoviridae	Plant	Coconut foliar decay virus (2e-09)	51
Mosquito VEM Geminivirus - SDWAP R	807	SD-WAP	-	-	Geminiviridae	Plant	Ageratum yellow vein virus (3e-09)	54
Mosquito VEM Geminivirus - SDWAP S - AG	574 - 2000	SD-WAP	-	-	Geminiviridae	Plant	Bean golden mosaic virus (1e-07 - 3e-14)	51 - 69
Mosquito VEM Geminivirus - SDWAP AF	593	SD-WAP	-	-	Geminiviridae	Plant	Blainvillea yellow spot virus (4e-05)	50
Mosquito VEM Geminivirus - SDWAP AG	451	SD-WAP	-	-	Geminiviridae	Plant	Pepper yellow leaf curl Indonesia virus (1e-05)	41
Mosquito VEM Geminivirus - SDWAP AH & AI	638 - 1074	SD-WAP	-	-	Geminiviridae	Plant	Soybean crinkle leaf virus (9e-11 - 8e-10)	46
Mosquito VEM Geminivirus - SDWAP AK	2150	SD-WAP	-	-	Geminiviridae	Plant	Squash leaf curl China virus (0.004)	59
Mosquito VEM Geminivirus - SDWAP AL	561	SD-WAP	-	-	Geminiviridae	Plant	Tomato leaf curl Java virus (2e-04)	50
Mosquito VEM Geminivirus - SDWAP AM -AO	530 - 939	SD-WAP	-	-	Geminiviridae	Plant	Tomato leaf curl New Delhi virus (2e-07 - 3e-11)	41 - 54
