# Supplementary material for: Broad Surveys of DNA Viral Diversity Obtained through Viral Metagenomics of Mosquitoes
Source: PLoS One. 2011 Jun 6;6(6):e20579. doi: 10.1371/journal.pone.0020579 (PMC3108952; doi:10.1371/journal.pone.0020579)
Supplement: Table S3 — Analysis of the contigs with amino acid identities (tBLASTx, evalue<0.001) to bacteriophages and insect viruses. (PDF) [file pone.0020579.s004.pdf]

Table S3

Analysis of the contigs with amino acid identities (tBLASTx, evalue<0.001) to bacteriophages and insect viruses.

Most significant matches from Genbank (tBLASTx, evalue < 0.001)	Mosquito SD-BVL virome			Mosquito SD-RB virome			Mosquito SD-WAP virome		
	Number of Contigs	Min of % a.a. identity	Max of % a.a. identity	Number of Contigs	Min of % a.a. identity	Max of % a.a. identity	Number of Contigs	Min of % a.a. identity	Max of % a.a. identity
**Invertebrates**									
Iridoviridae									
Chilo iridescent virus							4	41	54
Parvoviridae									
Aedes aegypti densovirus	2	63.64	95.24	1	100	100	1	100	100
Aedes aegypti Thai densovirus	3	58.33	81.25				1	88.29	88.29
Aedes albopictus densovirus	56	43.48	100	203	46.88	100	20	75	100
Anopheles gambiae densonucleosis virus	1	67.74	67.74						
Culex densovirus 0507JS11				1	88.24	88.24			
Culex pipiens densovirus	4	43.9	53.33						
Diatraea saccharalis densovirus	1	32.59	32.59						
Haemagogus equinus densovirus	65	44.83	100	84	40.54	100	6	94.94	100
Mythimna loreyi densovirus	2	43.75	47.13						
Toxorhynchites splendens parvovirus				1	95.24	95.24			
Poxviridae									
Amsacta moorei entomopoxvirus 'L'							1	44.74	44.74
unclassified									
Gryllus bimaculatus nudivirus	1	40	40	9	33.33	64.86			
Heliothis zea virus 1				1	40.38	40.38			
Oryctes rhinoceros virus	50	26.19	66.67	114	25	72.41	12	36.25	66.67
**Bacteria**									
Myoviridae									
Aeromonas phage 31	1	48.84	48.84						
Burkholderia phage phiE255	2	43.24	62.79				1	44.58	44.58
Campylobacter phage CPt10							1	62.86	62.86
Enterobacteria phage RB43	1	51.28	51.28						
Escherichia phage rv5	1	50	50						
Haemophilus phage Aaphi23	1	60.53	60.53				1	70.24	70.24
Iodobacteriophage phiPLPE							1	39.29	39.29
Pseudomonas phage LBL3	1	32.08	32.08						
Pseudomonas phage phiCTX	1	58.82	58.82						
Serratia phage KSP20	2	62.5	72						
Pseudomonas phage SN	1	70.59	70.59						
Cyanobacteria phage AS-1				1	89.74	89.74			
Podoviridae									
Burkholderia phage Bcep22	2	50	58						
Burkholderia phage BcepIL02	1	64.71	64.71						
Enterobacteria phage 13a	3	54.35	83.33						
Enterobacteria phage 285P	5	47.69	64.44						
Enterobacteria phage BA14	2	34.78	55.36						
Enterobacteria phage EcoDS1	6	50	82.76						
Enterobacteria phage epsilon15	1	70.27	70.27						
Enterobacteria phage K1F	1	72.73	72.73						
Enterobacteria phage N4							5	46.43	59.15
Enterobacteria phage phiV10	1	41.46	41.46						
Enterobacteria phage T3	1	43.14	43.14						
Enterobacteria phage T7	4	44.58	75.76						
Klebsiella phage K11	2	55.77	85.19						
Klebsiella phage KP32	6	52.5	61.36						
Kluyvera phage Kvp1	3	52.94	67.39						
Morganella phage MmP1	3	52.38	63.33						
Pseudomonas phage F116	1	70	70	1	80.43	80.43			
Pseudomonas phage gh-1	1	58	58						
Pseudomonas phage LIT1							4	36.59	53.49
Pseudomonas phage LUZ7							3	45.1	69.16
Salmonella phage phiSG-JL2	4	49.18	71.43						
Vibrio phage N4	1	44.07	44.07						
Vibrio phage VP2							1	31.82	31.82
Vibriophage VP4	2	50	54.05						
Yersinia pestis phage phiA1122	1	32.79	32.79						
Yersinia phage phiYeO3-12	2	62.22	70.37						
Yersinia phage Yepe2	3	50	71.43						
Acyrthosiphon pisum secondary endosymbiont phage 1				15	87.23	100			
Acyrthosiphon pisum secondary endosymbiont phage 2				6	93.02	100			
Enterobacteria phage ST64T				1	76.67	76.67			
Siphoviridae									
Burkholderia phage BcepNazgul	1	47.69	47.69						
Enterobacteria phage ES18	4	51.35	75.76						
Enterobacteria phage HK97	1	52.78	52.78						
Enterobacteria phage N15	2	38.78	57.45						
Enterobacteria phage TLS	1	53.49	53.49						
Flavobacterium phage 11b							4	45.33	55.26
Klebsiella phage phiKO2	1	85.29	85.29						
Phage phiJL001							1	24.19	24.19
Pseudomonas phage B3	4	55.1	68.75						
Pseudomonas phage F10	1	55.17	55.17						
Xanthomonas phage Xop411							1	43.37	43.37
