# Supplementary material for: Broad Surveys of DNA Viral Diversity Obtained through Viral Metagenomics of Mosquitoes
Source: PLoS One. 2011 Jun 6;6(6):e20579. doi: 10.1371/journal.pone.0020579 (PMC3108952; doi:10.1371/journal.pone.0020579)
Supplement: Table S4 — BLASTn analysis of the contigs with nucleotide identities to members of Papillomaviridae and Parvoviridae. (PDF) [file pone.0020579.s005.pdf]

Table S4

BLASTn analysis of the contigs with nucleotide identities to members of *Papillomaviridae* and *Parvoviridae* .

| Most significant matches from Genbank (BLASTn, evalue < 0.001) | Mosquito SD-BVL virome | | | Mosquito SD-RB virome | | | Mosquito SD-WAP virome | | |
|---|---|---|---|---|---|---|---|---|---|
| | Number of Contigs | Min % n.t. identity | Max % n.t. identity | Number of Contigs | Min % n.t. identity | Max % n.t. identity | Number of Contigs | Min % n.t. identity | Max % n.t. identity |
| **Animal** | | | | | | | | | |
| *Papillomaviridae* | | | | | | | | | |
| Human papillomavirus type 112 | | | | 3 | 71 | 91 | | | |
| Human papillomavirus type 23 | | | | 27 | 98 | 100 | | | |
| **Insect** | | | | | | | | | |
| *Parvoviridae* | | | | | | | | | |
| Aedes aegypti densovirus | 4 | 97 | 100 | 1 | 94 | 94 | | | |
| Aedes aegypti Thai densovirus | 1 | 95 | 95 | | | | | | |
| Aedes albopictus densovirus | 258 | 87 | 100 | 182 | 85 | 100 | 18 | 90 | 100 |
| Culex densovirus 0507JS11 | 1 | 100 | 100 | | | | | | |
| Diatraea saccharalis densovirus | 1 | 94 | 94 | | | | | | |
| Haemagogus equinus densovirus | 336 | 83 | 100 | 131 | 87 | 100 | 11 | 84 | 100 |
| Toxorhynchites splendens parvovirus | 2 | 85 | 90 | 4 | 84 | 89 | | | |
