# Supplementary material for: Broad Surveys of DNA Viral Diversity Obtained through Viral Metagenomics of Mosquitoes
Source: PLoS One. 2011 Jun 6;6(6):e20579. doi: 10.1371/journal.pone.0020579 (PMC3108952; doi:10.1371/journal.pone.0020579)
Supplement: Table S5 — Cross-BLASTn analysis of the mosquito metagenomes. A sequence was considered to be shared by two metagenomes if each sequence was the best BLASTn similarity for the other when the two metagenomes were compared with BLASTn. (PDF) [file pone.0020579.s006.pdf]

Table S5. Cross-BLASTn analysis of the mosquito metagenomes. A sequence was considered to be shared by two metagenomes if each sequence was the best BLASTn similarity for the other when the two metagenomes were compared with BLASTn.

Sample	SD-BVL	SD-RB	SD-WAP
SD-BVL	100.00%	12.00%	11.40%
SD-RB	12.00%	100.00%	5.21%
SD-WAP	11.40%	5.21%	100.00%
