# Supplementary material for: Broad Surveys of DNA Viral Diversity Obtained through Viral Metagenomics of Mosquitoes
Source: PLoS One. 2011 Jun 6;6(6):e20579. doi: 10.1371/journal.pone.0020579 (PMC3108952; doi:10.1371/journal.pone.0020579)
Supplement: Table S6 — Overview of the VEM methodology for obtaining viral metagenomes from mosquitoes. (PDF) [file pone.0020579.s007.pdf]

Table S6. Overview of the VEM methodology for obtaining viral metagenomes from mosquitoes.

1. Collection of mosquitoes
2. Homogenization
3. Removal of mosquito debris and cells by centrifugation and filtration through 0.45 µm syringe filter unit
4. Purification of viral particles by CsCl density-dependent centrifugation
5. Chloroform treatment
6. DNase I incubation
7. DNA extraction using CTAB/Formamide
8. Sequence-independent amplification using Phi29 DNA polymerase
9. DNA shearing
10. Linker-addition and PCR amplification of fragments
11. 454 pyrosequencing
12. Bioinformatic analyses
13. Confirmation by specific PCR
