# Supplementary material for: Broad Surveys of DNA Viral Diversity Obtained through Viral Metagenomics of Mosquitoes
Source: PLoS One. 2011 Jun 6;6(6):e20579. doi: 10.1371/journal.pone.0020579 (PMC3108952; doi:10.1371/journal.pone.0020579)
Supplement: Table S7 — Primers used in this study. (PDF) [file pone.0020579.s008.pdf]

Table S7. Primers used in this study

Genome/Sequence	Primers 5'to3'
Mosquito VEM Anellovirus - SDBVL A	GCATACGTACAATACTCCGCC
	GCAGAAGACTACGACCAGTGG
Mosquito VEM Anellovirus - SDBVL B	ATCTTAGGCATCTTGCGCCT
	ATGTTCGTGCCACCTCCTAG
Mosquito VEM Circovirus - SDBVL C	AGGAAGAAACGGGCGAAAAC
	TCTTTACAAGGTTCTAGGTGTGCTC
Mosquito VEM Circovirus - SDBVL D	TGCCACGAGAATTATCCAGA
	GCTGCCCATAAGACATCGTT
Mosquito VEM Circovirus - SDBVL E	GGTGAGGAAGTCGGTGAGAA
	ACAATACGTGACGTTCTGCTC
Mosquito VEM Geminivirus - SDBVL F	GATGGCGTCCCTCAACATC
	CCTTGGGCGATTGTTCATT
Mosquito VEM GeminiFungivirus - SDBVL G	TATGCAGATTGGCACTACCG
	TGGGTGCCTAATGATCTTGC
Mosquito VEM Anellovirus - SDRB A	GGTTACTGGTTGACCAGCGA
	CCAAGGATTTGACCTCTTCAG
Mosquito VEM Anellovirus - SDRB B	CTTGGTTGCTTGCAGACCA
	AAGGATTTGATCTTGGACGC
Mosquito VEM CircoNanoGeminivirus - SDRB AJ	AGAGCCTTCACAGTAGCGAA
	CTTCCCGGGTGCCATATAG
Mosquito VEM Anellovirus - SDWAP B	AGACCTCCAGGAGCAACAGA
	ATCTGAGGCTTCCCCATTTT
Mosquito VEM Densovirus - SDWAP	TGCGGACAACGGAAGCAACA
	TGCTGTGGTGATCCAAGTTGGTGT
